# Incidence of Reported Flu-Like Syndrome Cases in Brazilian Health Care Workers in 2020 (March to June)

**DOI:** 10.3390/ijerph18115952

**Published:** 2021-06-01

**Authors:** Ada Ávila Assunção, Emanuella Gomes Maia, Renata Jardim, Tânia Maria de Araújo

**Affiliations:** 1 Departamento de Medicina Preventiva e Social, Universidade Federal de Minas Gerais, Belo Horizonte 30130-100, Brazil; 2 Departamento de Ciências da Saúde, Universidade Estadual de Santa Cruz, Ilhéus 45662-900, Brazil; egmaia@uesc.br; 3 Departamento de Educação e Saúde, Universidade Federal de Sergipe, Lagarto 49100-000, Brazil; renatajardim@academico.ufs.br; 4 Departamento de Saúde, Universidade Estadual de Feira de Santana, Feira de Santana 44036-900, Brazil; araujo.tania@uefs.br

**Keywords:** health-care workers, flu-like syndrome, Brazil, SARS-CoV-2, COVID-19

## Abstract

Health care workers (HCWs) are at an increased risk of being exposed to COVID-19. This study aimed to characterize flu-like syndrome cases (FS) in HCWs notified in Brazil and compare them with FS cases in the general community (GC). In the Brazilian protocol, FS corresponds to a suspected case of COVID-19. The manuscript analyzed cases of FS in five Brazilian states, estimating the incidence rates of cases of FS and clinical and epidemiological characteristics. Registered cases (March to June 2020) totaled about 1,100,000 cases of FS. HCWs represented 17% of the registers, whose incidence was 20.41/100 vs. 2.15/100 in the GC. FS cases in HCWs concentrated the highest percentages in the age group of 30 to 49 years (65.15%) and among the nursing staff (46.86%). This study was the first interstate evaluation in Brazil to estimate suspected cases of FS by COVID-19 in HCWs. In order to control the spread of viral respiratory infections in HCWs, including COVID-19, it is necessary to review the management of health information to identify who they are, how many they are, and to what situations these workers are most frequently exposed, as well as in what professions they have. This information can guide specific, practical, and far-reaching actions.

## 1. Introduction

The protection of HCWs is crucial to face the pandemic crisis [1,2,3,4]. The availability of beds, adequate number of fans, oxygen, medications, a comprehensive testing scheme, monitoring of the genetic diversity of virus strains, and other resources will not bring the desired effects without sufficient workforce staff and safe and protected conditions in the work environment [5]. Community health workers, nurses, doctors, physiotherapists, pharmacists, laboratory technicians, all other support agents, and professionals working in long-term institutions, who provide health care to citizens, are unrelenting in their efforts to face the pandemic crisis [2].

HCWs are working in unsafe conditions. Accounting for 3% of the population in most countries, HCWs comprise 14% of COVID-19 cases reported to the World Health Organization (WHO) [1]. More than 7000 HCW deaths have been reported as a result of COVID-19 globally [1]. These data show the vulnerability of HCWs [3,6], who can become vehicles of transmission to their patients and the community [7,8]. In Brazil, where the 14,441,563 COVID-19 infection cases have already caused more than 395,000 deaths (April 2021), there is little information about COVID-19 cases among Brazilian HCWs during the current pandemic. The mean transmission potential (R0) of COVID-19 is three times greater in Brazil when compared to other countries [9]. The actual number of COVID-19 cases is seven times greater than the number of registered cases [10]. Despite the fact that, according to the Brazilian protocol, all symptomatic HCWs must be tested, HCW-specific COVID-19 data and estimates are currently unavailable. The problem of accessing the tests is recognized [11]. It is worth mentioning that the Brazilian Ministry of Health (BMH) assumes this reality in the protocol itself, as it includes the recommendation of removing from work symptomatic HCWs that have not been tested [12]. Additionally, there are some insurmountable difficulties in calculating the proportion of people infected by occupational subgroup and providing other relevant information for controlling exposure. For example, the demographics characteristics of the workforce are not sufficiently known in Brazil. Data on COVID-19 cases and deaths have been reported using crude rates, thus preventing the identification of the most affected population groups and subgroups. It would be necessary to calculate the proportions of infected, using the population size as the denominator [13], since infection, disease severity, intensive care admission, and death vary by gender and age group [14]. All the situations mentioned contribute to the lack of a structured, transparent, and comprehensive surveillance system for Brazilian HCWs [11].

To explore the situation of HCWs, this study analyzed official data available on the Brazilian Ministry of Health’s portal [15] to describe the clinical and epidemiological characteristics of the first 1,100,000 reported flu-like syndrome (FS) cases, the most common manifestation of COVID-19 [16,17]. Knowledge of the occurrence profiles through the description and correlation of the clinical and individual characteristics of the suspected cases and the geographic location is urgent for the management of exposure to the risk of HCW infection and, therefore, for coping with the pandemic. The principal aim of this investigation was to characterize reported FS cases in HCWs and compare them to FS cases in the general community (GC).

## 2. Materials and Methods

### 2.1. Study Design and Population

This manuscript was based on the e-SUS Notifica system public dataset [18], accessed through the website (https://opendatasus.saude.gov.br/dataset/casos-nacionais (accessed on 27 June 2020)) which has been in force since March 2020.

The study focused on cases of FS in the Brazilian adult population (20 years or older) notified in the states with the largest number of municipalities in each of the five macro-regions of the country: Minas Gerais (853 municipalities, southeast region), Rio Grande do Sul (497 municipalities, south region), Bahia (417 municipalities, northeast region), Goiás (246 municipalities, mid-west region) and Pará (144 municipalities, north region)—a total of 2157 municipalities. This manuscript considered the data recorded and updated until 27 June 2020, covering the first months of the pandemic in the country.

### 2.2. Data Organization and Variable

The relationship between occupational exposure among HCWs and FS is the focus of this study. Therefore, whether or not the subject is a HCW and the registration of the occupation of these professionals following the Brazilian Occupations Code (CBO) were analyzed. Based on the classification of healthy occupations suggested by the World Health Organization (WHO Atlas) [19], the analysis considered seven occupational subgroups of the health services: doctors; nurses and nurse technicians; dentists and dental technicians; biochemists and laboratory technicians (appointed as “laboratory”); community health agents; physical therapists, occupational therapists, psychologists, and other mental health workers (appointed as “therapists, “psychologists”); higher education professionals, technical education professionals, administrative, janitorial services, and others (appointed as “administrative and general services staff”).

A set of three sociodemographic variables and five clinical and epidemiological variables complemented the analyzes: sex (male/female), age group (20 to 29 years/30 to 49 years/50 to 69 years/70 years or older), race/ethnicity (White/Pardo/Black/Asian/Indigenous), test status (requested/collected/completed), test type (rapid antibody test/rapid antigen test/RT-PCR), test result (negative/positive), case evolution (cure or home treatment/hospitalization and admission to an intensive care unit (ICU)/death) and final classification (discarded/laboratory confirmation/clinical-epidemiological confirmation).

### 2.3. Data Analysis

The analysis initially focused on the absolute and relative distribution of cases of FS Brazil according to the sociodemographic profile (sex, age group, race/ethnicity and states), considering HCWs and the GC.

In sequence, the incidence rates (IR) for cases of FS were calculated, considering the clinical and epidemiological data for each main group (HCWs and the GC). The formula IR = A/B × 100 was used for this calculation. For HCWs, A was the number of FS cases notified among the HCWs, and B was the total number of HCWs registered in the National Registry of Health Facilities (CNES) [20] of the five states analyzed in the study (n = 911,560). For the GC, A was the number of FS cases notified in the GC, and B was composed of the total number of adults in the GC from these five states, according to the 2010 Demographic Census projected for 2020 (n = 42,312,269) [21]. In that last calculation, the number of health professionals registered in CNES was subtracted from the total number of adults from the GC. The study also analyzed the relative distribution of cases of FS in Brazil according to occupational subgroups stratified by sex, age group, and state.

To estimate incidences, the data related to the numerator come from e-SUS Notifica-an electronic database of national scope, consolidated based on data from states and municipalities, which identify and record all cases of SF in the Brazilian territory. Notification of the SF case is mandatory. Therefore, the expectation is that all cases that meet the notification criteria (flu-like syndrome) will be identified and registered in this system [22,23]. As the data depends on registration in health services, there are potential biases of underreporting due to access to these services. Thus, despite the scope and capillarity of e-SUS Notifica, problems in identifying cases cannot be ruled out.

Microsoft Office Excel 2010 (Microsoft Corp., Redmond, WA, USA) was used to analyze the consistency of the databases for the five selected states, and the statistical software Stata (version 14.2) was used to create the final database, as well as the organization and analysis of the data.

The analysis of secondary data for public access and use (such as those extracted from Datasus) does not require the approval of the Research Ethics Committee according to CNS Resolutions No. 466/2012 [24] and 510/2016 [25].

## 3. Results

In the analyzed period, more than 1,100,000 cases of FS were reported in five Brazilian states, and more than half of the cases were female (54.51%), aged between 30 and 49 years old (52.61%), with higher percentages in Minas Gerais and Bahia (31.63% and 26.06%, respectively) (Table 1). HCWs represented 17% of the notified cases. In this group, there is a greater concentration of women (75.20% vs. 50.22% in the general community) and adults in the age group of 30 to 49 years (65.15% vs. 49.90% in the GC). Bahia registered the highest proportion of cases in HCWs (38.91%) compared to the other states. Minas Gerais stood out with the highest proportion of cases registered in the GC (32.40%) in relation to the other states (Table 1). Although there is a specific field for recording race/ethnicity in the form, there were missing values in 100% of the records (data not shown).

**Figure 1:**
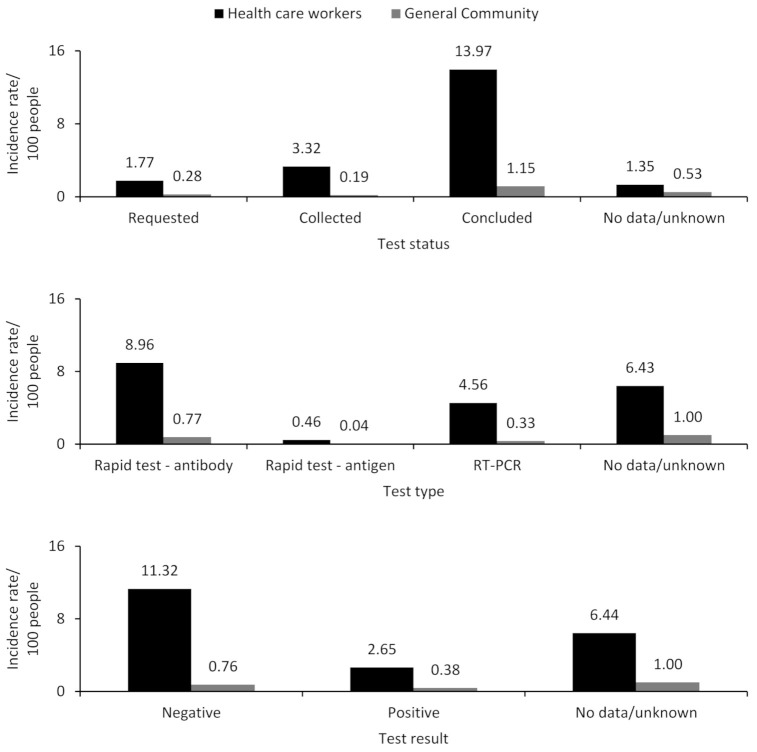

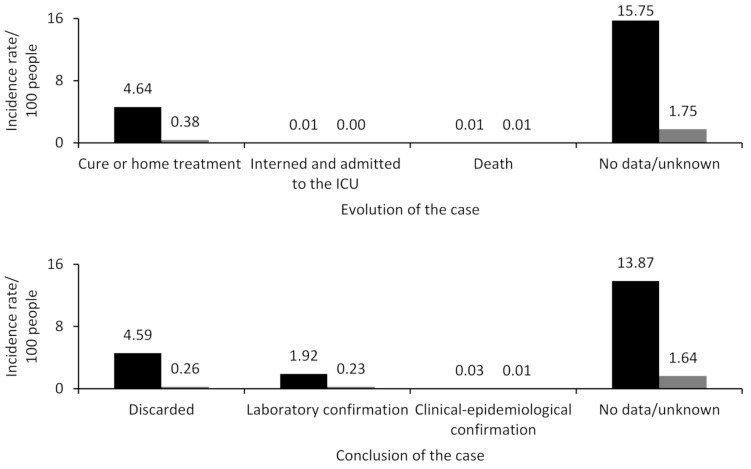
Incidence rates of clinical and epidemiological data of cases of flu-like syndrome in Brazil ^a^, considering health care workers (HCWs) and general community. DATASUS, Population Census ^b^ and National Register of Health Establishments, 2020. ^a^ It is equivalent to the total number of cases in the largest states of each macro-region in the country, considering the number of municipalities. The data was updated until 27 June 2020. ^b^ The values of the 2010 census were adjusted considering the population projection for 2020 of the IBGE. ICU: intensive care unit.

The comparison of the incidence rate between the two main groups shows relevant differences, being 10 times higher in the HCWs in relation to the incidence observed in the GC (global incidence rate of 20.41/100 people vs. 2.15/100 people) (Figure 1). For all variables, the incidence rate was higher in the HCW group, with an emphasis on the concluded test status (13.97/100 people vs. 1.15/100 people), rapid antibody test performed (8.96/100 people vs. 0.77/100 people), negative test result (11.32/100 people vs. 0.76/100 people), evolution of the case for cure or home treatment (4.64/100 people vs. 0.38/100 people), and conclusion of the case as discarded (4.59/100 people vs. 0.26/100 people) (Figure 1). The poor completeness of data on clinical and epidemiological characteristics was also greater among the HCWs, reaching, on average, seven times more occurrences of unknown/no information when compared to the general community group (average rate of 7.41). Failures to register case follow-up were evident, whether referring to the evolution or conclusion of it (15.75/100 people for HCWs vs. 1.75/100 people for the general community, and 13.87/100 people for HCWs vs. 1.64/100 people for the general community, respectively) (Figure 1).

**Figure 2:**
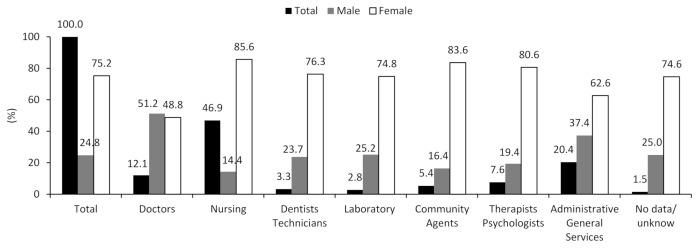

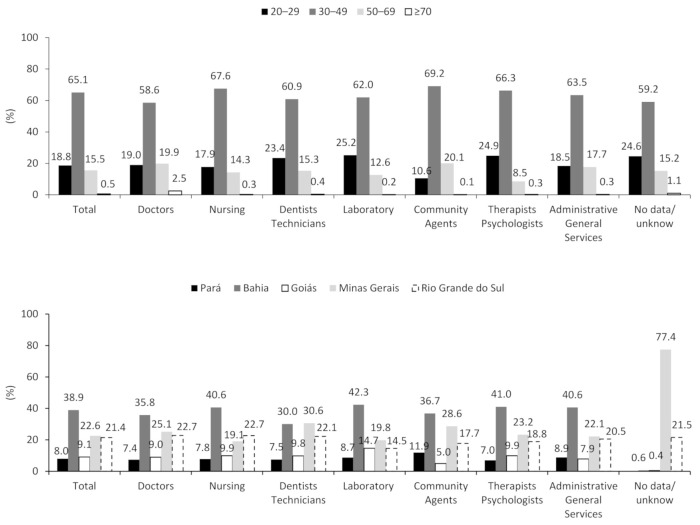
Relative distribution of occupation of health professionals notified as cases of flu-like syndrome in Brazil ^a^, according to sex, age group, and states. DATASUS, e-SUS Notifica. ^a^ It is equivalent to the total number of cases in the largest states of the country, considering the number of municipalities. The data updated until 27 June 2020. Note 1: percentage was estimated considering the total (N = 186,044) of reported cases. Note 2: the total of missing or unknown data for the sex variable among health professionals was 11 cases. Therefore, for the sex variable, N = 186,033.

As expected, due to the predominance of women in the healthcare sector, the female percentage represented 75.2% of total cases, with higher proportions in the occupations of nursing and community agents (85.6% and 83.6%, respectively). Only among doctors did we observe a higher percentage of males (51.2%). Of the total, almost half of the HCWs notified with FS were nursing professionals (46.9%); followed by administrative general services professionals (20.4%). Doctors occupied the third position (12.1%). The proportion of unknown data for the occupation variable in the HCW group was low (1.5%) (Figure 2). Regardless of occupation, the 30-to-49-year age group was the most prevalent in the HCW group, representing 65.1% of cases. The distribution of percentages by age group showed a higher percentage of doctors in the 70-years-and-older group (2.5%) when compared to other occupations in the same age group. In the youngest age group (from 20 to 29 years), laboratory professionals predominate (25.2%) (Figure 2). Notifications in the HCW group were more prevalent in the states of Bahia, Minas Gerais and Rio Grande do Sul (38.9%, 22.6%, and 21.4%, respectively). The high proportion of missing or unknown data on occupation in the HCW group in the state of Minas Gerais stands out (77.4%) (Figure 2).

## 4. Discussion

The distribution of cases of FS was described in a sample of the Brazilian adult population aged 20 and over, using data from 2020 (March to June) in the e-SUS Notifica system [18]. We identified a tenfold higher incidence of reported FS cases in the HCWs by contrasting the incidence in the GC. It is worth noting FS’s high incidence rate for completed test status, type of rapid antibody test, and negative test result among HCWs. Nursing professionals, administrative general services professionals, and doctors were the most prevalent occupations among the registered cases. Female sex, age group 30 to 49 years, and the states of Bahia and Minas Gerais stood out, regardless of the type of occupation.

The results presented on FS notifications were facilitated by the e-SUS Notifica system, a system implemented at the onset of the COVID-19 epidemic to record information about FS and SARS. This system planned to cover the national territory aims to collect fundamental data for understanding the COVID-19 epidemiological behavior. To this end, variables on the testing, clinical evolution, and occupation of the individual treated with suspected infection were included. On the one hand, the results confirm the capillarity of the e-SUS Notifica system and the SUS. On the other hand, the problems identified in the quality of information indicated failures in the management of this information system, due to the failure to complete the fields of ethnicity, occupation, and case development. Therefore, caution is recommended when analyzing the results obtained. Apparently, these flaws are not just due to unpreparedness in data collection.

The pandemic infection tracking and monitoring system is inefficient. The first recorded case of COVID-19 in Brazilian territory occurred on 26 February 2020 [26]. The epidemic spread rapidly, with a marked rate of transmission compared to other countries. According to statistics from the Imperial College London, Brazil already ranked second in the number of cases in May of that year. Notwithstanding this, to date, no specific federal programs are available for monitoring and controlling infection for HCWs. This gap has an important impact on epidemiological surveillance, reliable data availability, and, above all, coping with the pandemic. Moreover, laboratory testing has been reserved for SARS cases [27].

The high incidence rate of negative test results in HCWs compared to the GC is possibly an effect of the test access difference since HCWs are considered a vulnerable group [12]. However, to the detriment of the reverse transcription-polymerase chain reaction (RT-PCR) test, the rapid antibody test type’s high rate suggests a late referral for testing. This result did not differ between the populations of each analyzed state. Specifically, for HCWs, the Brazilian Health Ministery´s protocol recommends RT-PCR testing after three to seven days and rapid serological testing (IgM/IgG) after the eighth day of onset of symptoms [28]. It is appropriate to speculate on the late approach of symptomatic HCWs based on these recommendations that consider the period of symptoms. The mean between the onset of symptoms and the test for COVID-19 was 10.2 days [22] in the federal district and capitals of the Brazilian states. The high incidence rate of registered FS cases in HCWs compared to the GC can also be attributed to the access criteria.

Proportionally, we observed more women in the sample of notified cases of FS. These results were expected [29,30]. Despite this, it is essential to highlight that the sexual division of labor and its social valorization system establish gender inequalities, which, in turn, influence the different vulnerabilities of women and men to infection, exposure to pathogens and treatments received, and vary according to professional categories [29,31].

In the HCW group, the reported FS cases are concentrated in the 30 to 49 age group. While the manifestation of COVID-19 symptoms is directly proportional to advancing age, in Egypt [32,33], the mean age of infected HCWs coincides with the age group identified in this study. Caution in this comparison is required because our results refer to reported FS cases of unknown etiology. In Egypt, the cases were tested positive for COVID-19. Future investigations should speculate on the age discrepancies in the distribution of cases of flu-like respiratory syndromes in HCWs.

This study did not describe the distribution of suspected or confirmed cases according to race/ethnicity, due to the denied information. Information deficit is a sign of the structural racism of our society, which maintains racial inequalities [34]. The absence of information about race/ethnicity in the forms that feed social information systems is so widespread that it provoked, through ministerial decree, the obligation of the procedure in instruments of information collection in health services [33]. However, as seen, the establishment as mandatory was not enough. Structural racism generates behaviors, beliefs, and prejudices underlying preventable inequalities between social groups based on race or ethnicity.

Nursing staff stand out in the ranking of the distribution of cases among HCWs according to the occupational subgroup, representing almost half of the relative distribution of suspected FS cases reported, consistent with findings from other studies. This occupational subgroup is constantly exposed to viral materials due to the tasks performed in the wards or the frontline of the first procedures in urgent situations, in constant contact with the patients’ relatives and colleagues [30]. A relevant result to highlight is that professionals performing tasks that, in theory, do not require dealing directly with patients occupied the second position in the number of affected. In France, the incidence rate of symptomatic COVID-19 infection also varied when comparing occupational subgroups [35]. However, some controversies are observed in the literature. For example, in England [36] and China [37], no differences were found in positive testing when comparing those exposed and unexposed to direct contact with the patient. Two hypotheses can be raised to explain this apparent controversy. The first relates to community transmission, which can vary from one group or one establishment to another. HCWs cross health service environments with a high concentration of viral materials and the domestic and community environments. Identifying genetically distinct strains in HCWs from the same establishment confirmed community transmission in this occupational group in the U.S. [8] and the Netherlands [7]. The second hypothesis about the distribution of cases according to the occupational subgroup is associated with the differences related to the professional qualification of each subgroup, which concerns characteristics such as training, social recognition, category organization, and political power of its corporate and representative institutions.

The limitations of this study should be highlighted. The deficient data source [9,19] raises caution when assessing and interpreting the results. While admittedly a powerful tool, DATASUS, which hosts numerous Brazilian health information systems, including e-SUS Notifica, has been criticized regarding the quality of processed and stored data [38]. For example, the lack of completed ethnicity/skin color field data is noteworthy. For this reason, as already mentioned, it was not possible to explore the hypothesis about racial disparities in the distribution of FS cases. There was also a loss of information on the evolution and conclusion of the notified cases and a lack of homogeneity in the procedures for registering notifications between the analyzed states. Possibly, the different information management systems, from the type of training of the responsible technician to the completion mode (manual, electronic, or mixed) [39] have influenced our results. Except for Minas Gerais, the occupation field for non-HCWs was not completed. This information would help in the planning of policies to address the viral pandemic for at least two reasons: workplaces are a source of disease spread to the entire population and a source of exposure for those who work there [40]. The inconsistencies observed suggest clues for the action of e-SUS Notifica managers.

Despite these limitations, this study produced a set of original data revealing part of a reality hitherto unknown. In unprecedented fashion in the country, the characteristics of FS cases in HCWs were explored compared to the GC in the first months of the COVID-19 pandemic. Sociodemographic characteristics (gender, age group, and federal state) were considered in the analyses in HCWs and the GC and the stratified analyses by occupational subgroups of the health services. It is worth highlighting the strategy of searching for information in the national demographic census [21] and in the register of health establishments in the country [20] to produce the denominators. This strategy produced proportions [13] instead of crude rates. The results confirm the relevance of HCWs’ exposure to infection risk [1,2,3,4,6,11,30,35,37,41]. We avoided producing localized results by studying a state in each region of the country. In this sense, this knowledge will probably help develop policies to protect HCWs from the risk of exposure to COVID-19 and improve the management of health information systems.

Efforts will be needed to articulate sectoral policies to strengthen health professionals’ protection actions. These policies depend on detailed information such as: how many there are, what situations these workers are most frequently exposed to, and the most vulnerable occupations. The type of contact with the public, maintenance and hygiene of places, access to facilities for personal care, training in the use of personal protective equipment (PPE), and availability of appropriate equipment, among other factors, probably vary in quality and quantity between occupations and different establishments. As these characteristics must be well-known for the definition of adequate and effective measures to protect workers, it is recommended to implement occupational surveillance systems linked to epidemiological surveillance systems. Joint actions of these systems will allow the follow-up and monitoring of the evolution of the pandemic and the definition of the appropriate measures [6,40,41].

The resolution of the deficits and difficulties mentioned above is a crucial challenge for facing and overcoming the current health crisis. In this context, issues related to the demand for the agile dissemination of indicators to society and public managers are high-lighted [42]. In this sense, efforts at standardization and definitions in the agencies where the data are collected and recorded are desirable, and the training of the technical teams responsible for the processing and disseminating information.

Finally, investigations into occupational differences deserve more encouragement. Clear and accurate information on the status of occupational exposures and their impacts are valuable inputs for occupational and epidemiological surveillance, which, in turn, are crucial for pandemic control.

## 5. Conclusions

This study reported a greater notification of FS cases in HCWs compared to the general community. Poor quality information, on the one hand, and the lack of a transparent and comprehensive occupational surveillance system, on the other hand, advise against further interpretations of the results described. However, these results converge with the literature. As such, they are valuable clues for evaluating existing systems and contributing to future research designs.

## Figures and Tables

**Table 1:** Absolute and relative distribution of cases of flu-like syndrome in Brazil ^a^, considering health care workers (HCWs) and general community. DATASUS, e-SUS Notifica, 2020.

Variables	Total ^b^		HCWs		General Community		*p*-Value ^c^
	*n*	%	*n*	%	*n*	%	
Sex							
Male	501,179	45.48	46,123	24.79	442,022	49.77	*p* < 0.001
Female	600,623	54.51	139,910	75.20	446,010	50.22	
No data/unknown	104	0.01	11	0.01	19	0.00	
Age group (years)							
20–29	244,101	22.15	34,885	18.75	202,984	22.86	*p* < 0.001
30–49	579,704	52.61	121,206	65.15	443,173	49.90	
50–69	221,189	20.07	28,914	15.54	187,142	21.07	
70 or older	56,912	5.16	1039	0.56	54,752	6.17	
States							
Pará	163,377	14.83	14,961	8.04	147,014	16.55	*p* < 0.001
Bahia	287,179	26.06	72,398	38.91	211,064	23.77	
Goiás	109,194	9.91	16,882	9.07	90,212	10.16	
Minas Gerais	348,586	31.63	41,965	22.56	287,701	32.40	
Rio Grande do Sul	193,570	17.57	39,838	21.41	152,060	17.12	
Total	1,101,906	100.00	186,044	100.00	888,051	100.00	

^a^ It is equivalent to the total number of cases in the largest states of each macro-region in the country, considering the number of municipalities. The data updated until 27 June 2020. ^b^ The total column does not equal the sum of cases between health professionals and the general community. Of this total of notified cases, 27,811 did not have a data record of being or not being a health professional. ^c^ The *p* values were calculated considering only the valid data for each variable. Among the sociodemographic variables, only sex had missing data.

## Data Availability

**e**-SUS Notifica data are publicly available. Our analysis code is available online.
